# Polystyrene Microplastic Exposure Adversely Affects Oocyte Quality and Ovary Health Status in *Mytilus galloprovincialis*

**DOI:** 10.3390/ijms27135817

**Published:** 2026-06-27

**Authors:** Teresa Chianese, Mariachiara Galati, Tiziana Cappello, Maria Maisano, Sabrina Balsamo, Annamaria Locascio, Luigi Rosati, Rosaria Scudiero

**Affiliations:** 1 Department of Biology, University Federico II, 80126 Napoli, Italy; teresa.chianese2@unina.it (T.C.); sabrina.balsamo@unina.it (S.B.); luigi.rosati@unina.it (L.R.); 2 Department of Chemical, Biological, Pharmaceutical and Environmental Sciences, University of Messina, 98166 Messina, Italy; mariachiara.galati@unime.it (M.G.); maria.maisano@unime.it (M.M.); 3 Department of Biology and Evolution of Marine Organisms, Stazione Zoologica Anton Dohrn, 80121 Napoli, Italy; annamaria.locascio@szn.it; 4 Centro Interdipartimentale di Ricerca “Ambiente” (CIRAM), University Federico II, 80135 Napoli, Italy

**Keywords:** polystyrene microplastics, ovaries, metabolomics, reproduction, mussels

## Abstract

Microplastics pose a growing threat to marine ecosystems as they can accumulate in aquatic organisms, interfering with essential physiological processes including reproduction. This study analyzed the effects of short-term exposure (48 h) to two concentrations (0.5 and 1 µg/mL) of 5 µm diameter polystyrene microplastic bead particles in female *Mytilus galloprovincialis* mussels, a bioindicator species of the Mediterranean Sea. Histological analyses revealed progressive oocyte degeneration and the development of hypertrophic PAS-positive mucous cells, indicative of a stress response, in a dose-dependent manner. Changes in hemocyte classes, which are major effectors of bivalve immunity, were evidenced by the May–Grünwald Giemsa reaction. Biochemical data showed that microplastics increased levels of stress proteins, such as HSP70 and p53, and altered the composition of ovarian glycoproteins. Metabolomic analysis based on ^1^H NMR spectrometry revealed significant alterations in metabolites involved in energy (glucose, glycogen, and malonate) and amino acid (branched-chain amino acids arginine, glycine, glutamate, histidine, betaine, and choline) metabolism, suggesting impairment to bioenergetic and antioxidant pathways. Overall, these results suggest that even short-term exposure to polystyrene microplastic beads can alter the ovarian function and metabolism of female *M. galloprovincialis*, highlighting their vulnerability.

## 1. Introduction

Plastic debris represents a modern form of abiotic stress for ecosystems [1]. These small pieces of plastic can have different shapes (spheroid, fragment, fiber), sizes and can be made of different polymers [2]. Microplastics (MPs, <5 mm) form through the degradation and weathering of larger plastic items, entering the environment as mismanaged or improperly disposed of waste [3]. They can also be intentionally made and added to products to enhance certain properties, such as microbeads added to personal care and household products for their scrubbing effect [4]. MP pollution has reached every corner of the planet; evidence shows that MPs enter the food chain, particularly in marine environments, where they accumulate as they are not blocked by normal water treatment plants [5,6].

In coastal marine areas, filter-feeding animals, such as bivalve mollusks, are particularly engaged. By filtering the water, mollusks introduce these microparticles, which not only cause various damages to the filtering system but are also mistaken for food by the animals, giving them a false sense of satiety [7,8]. Nutrient deficiencies can further deteriorate the overall health of these animals [9].

The Mediterranean Sea is a semi-enclosed sea with a very slow and limited water exchange. For this reason, it tends to become easily polluted. In recent years, it has also been experiencing an unusual rise in water temperature, leading to its acidification [10]. Recent studies on the adverse effects of MPs and nanoplastics (NPs, <1 µm) pollution in mollusks have been performed on the Mediterranean species *Mytilus galloprovincialis*. These studies have shown how both MPs and NPs can accumulate in animals, raising concerns about the potential risk of their intake for humans through mussel consumption [11,12]. Indeed, various adverse effects have been observed in *M. galloprovincialis* tissues following exposure to MPs and NPs, including changes in immune response, cellular stimulus response, protein interactions, apoptosis, and cell death [13,14]. Organs such as the digestive gland and gills exhibit morphological and molecular alterations [15,16], as well as metabolic disorders [17].

More recently, given the fundamental importance of reproduction for species survival, the potential reproductive toxic effects of MPs have also been investigated in *M. galloprovincialis* [18,19]. Studies conducted on adult males during the breeding season showed that polystyrene MPs, upon reaching the testis, alter the structure of the sperm cyst in a dose-dependent manner, disrupting cellular interactions between germ cells. In addition, the occurrence of a pro-inflammatory state and an oxidative stress condition were also demonstrated, supported by metabolomics. Interestingly, MPs also disrupt the proper chromatin folding of spermatozoa, suggesting that, in addition to hindering gametogenesis, they may also impact the fertilizing capacity of these cells [18,19].

The aim of this study is to assess the condition of the female gonads following a 48-h exposure to two different concentrations of 5 µm diameter polystyrene MP beads, replicating the conditions used in previous research on males. In females, the general condition of the animals is much more crucial than in males for producing good-quality gametes. This is because the nutrients must be accumulated in the oocytes to ensure the early stages of embryonic development, up to the larval stage. Therefore, a multi-biomarker approach was herein employed, including histological evaluation of the investigated tissue using Hematoxylin and Eosin staining and the application of histochemical methods to assess the immune response, such as May–Grünwald Giemsa and Schmorl’s stain. Consistently with the previous study conducted on male gonads [19], Western blot analysis was also performed, to evaluate the level of proteins involved in cellular stress like HSP70 and p53, as well as analysis of the composition of glycoproteins in the ovary. Additionally, for a more comprehensive overview of the potential MP-induced biochemical alterations at cellular level, the -omics technique based on ^1^H NMR spectrometry was applied to assess the metabolic state of female gonads.

## 2. Results

### 2.1. Morphological and Histological Observations of the Mussel Ovary 

**Figure 1:**
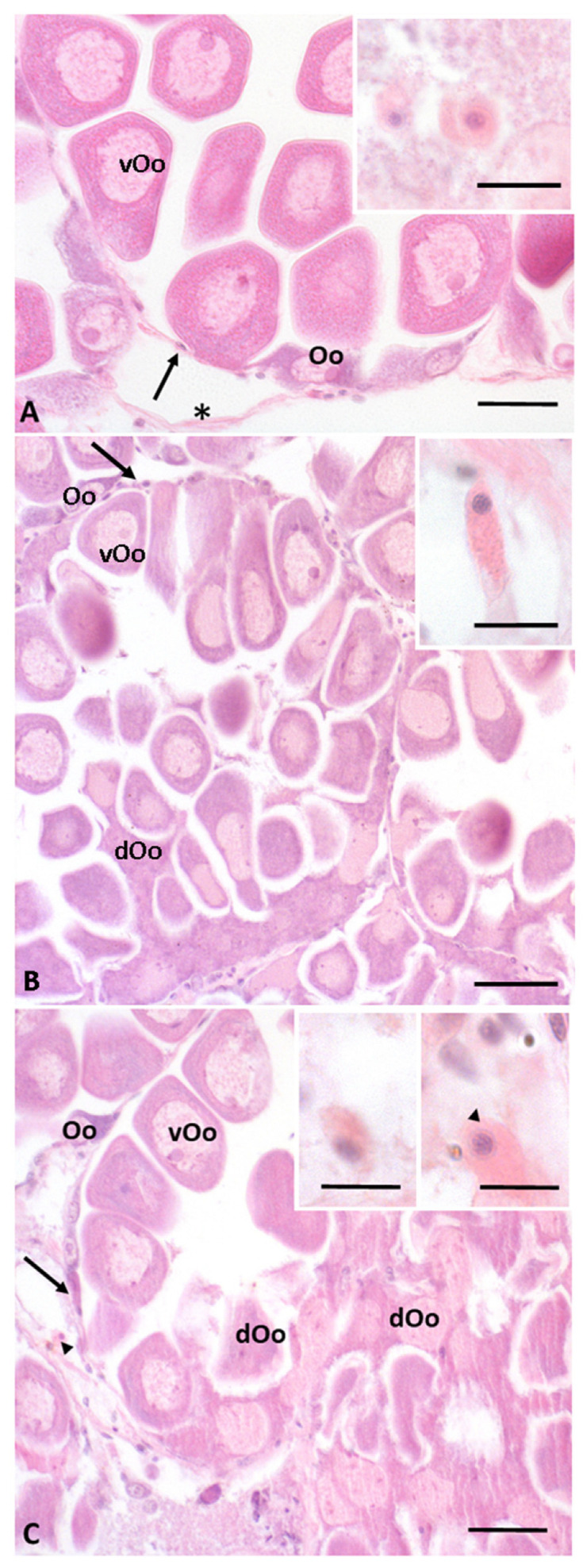
Hematoxylin–Eosin staining of gonad sections from *M. galloprovincialis* females. (**A**) Control: intact ovarian follicles rich in vitellogenic oocytes (vOo) ready for release are evident and oogonia located along the wall of the follicle (Oo). (**B**) Animals treated with 0.5 µg/mL of MPs: both some intact ovarian follicles rich in vitellogenic oocytes ready for release and degenerating oocytes (dOo) are evident. (**C**) Animals treated with 1 µg/mL of MPs: a greater number of degenerating oocytes are evident. Inserts: hemolymph cells present in the interstitial connective tissue; Arrows: follicles; Arrowheads: hemocytes; Asterisks: connective cells. Bars correspond to 50 µm, except for the inserts, which correspond to 10 µm.

*M. galloprovincialis* females of the control group showed normal ovarian organization, consistent with the reproductive period (January–March) [20]. In particular, the ovarian follicles were intact and contained vitellogenic oocytes with a typical pear shape, ready for release (vOo, Figure 1A). In animals exposed to 0.5 µg/mL of MPs (Figure 1B), both intact ovarian follicles with vitellogenic oocytes ready for release and follicles containing oocytes in the process of degeneration (dOo, Figure 1B) were observed. The latter, probably about to undergo apoptosis, can be recognized by the irregular shape of the nucleus and the characteristic cell shrinkage that precedes the apoptotic process. In animals treated with 1 µg/mL of MPs (Figure 1C), an apparent increase in the number of ovarian follicles containing degenerated oocytes was observed, compared to both controls and animals exposed to the lower concentration. In addition, a greater intensity of degenerative phenomena was detected, with evident alterations in both cytoplasmic and nuclear morphology of the oocytes, suggesting that exposure to higher concentrations of MPs amplifies cellular stress and the risk of ovarian apoptosis.

**Figure 2:**
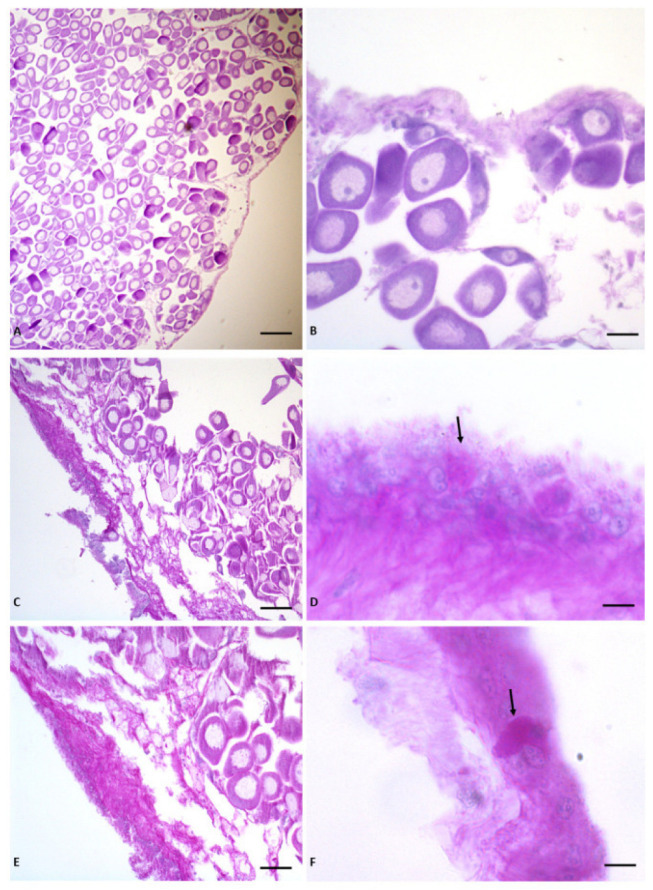
PAS of *M. galloprovincialis* ovary sections. (**A**,**B**) Control: no PAS-positive cells were observed. (**C**–**F**) Animals treated with 0.5 µg/mL (**C**,**D**) and 1 µg/mL (**E**,**F**) MPs: hypertrophic PAS-positive mucous cells are evident (arrow). The bars correspond to A = 100 µm, B = 30 µm, C = 60 µm, D–F = 10 µm, E = 50 µm.

A more detailed analysis of the ovarian sections performed using the periodic acid-Schiff staining (PAS), showed differences between control and MP-treated samples. In the controls (Figure 2A,B), no PAS-positive cells were found, indicating the absence of significant accumulations of glycoproteins in the observed areas. In contrast, in animals exposed to 0.5 µg/mL of MPs (Figure 2C,D), a marked presence of PAS-positive mucous cells was detected, located mainly in the peripheral regions of the gonad. These mucous cells appeared hypertrophic, suggesting an increase in glycoprotein synthesis and secretion, in response to the exposure to the contaminant. Similar and more pronounced effects were observed in specimens treated with 1 µg/mL of MPs (Figure 2E,F), with hypertrophic PAS-positive mucous cells present in the peripheral areas of the ovary, confirming a dose-dependent response to MPs exposure.

### 2.2. Histochemical Evaluation of the Mussel Ovary

The May–Grünwald Giemsa reaction allowed to differentiate three main classes of hemocytes. 

**Figure 3:**
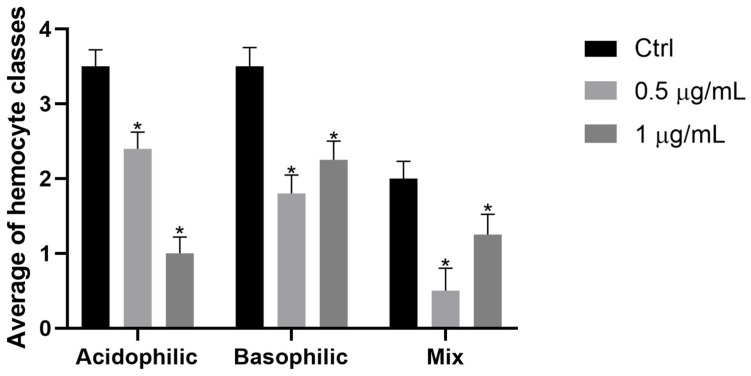
Graph showing abundance of the three different hemocyte classes in female gonads of *M. galloprovincialis* in control animals and after exposure to 0.5 and 1 µg/mL of PS MPs. Statistical analysis was performed by two-way ANOVA, considering treatment (Ctrl, 0.5 µg/mL, 1 µg/mL) and hemocyte class (acidophils, basophils, and mix) as factors, followed by the Holm–Šídák post hoc test for multiple comparisons between treatments and the control group within each cell class. Statistical significance was set at *p* < 0.05 and indicated with asterisks (*).

Specifically, in the female gonads of the control group, hemocytes were identified as (i) granular, containing a large nucleus in abundant acidophilic cytoplasm, (ii) agranular, distinguishable by their smaller size compared to the previous ones and by having rounder contours surrounding a small nucleus and basophilic cytoplasm, and (iii) mixed, with small hemocytes that responded positively to the reaction as both basophils and acidophils. It is noteworthy that all the detected hemocyte classes significantly decreased compared to what was found in the tissues of the control organisms. The graph in Figure 3 illustrates the values of the three hemocyte classes in the control gonads and following exposure to MPs.

**Figure 4:**
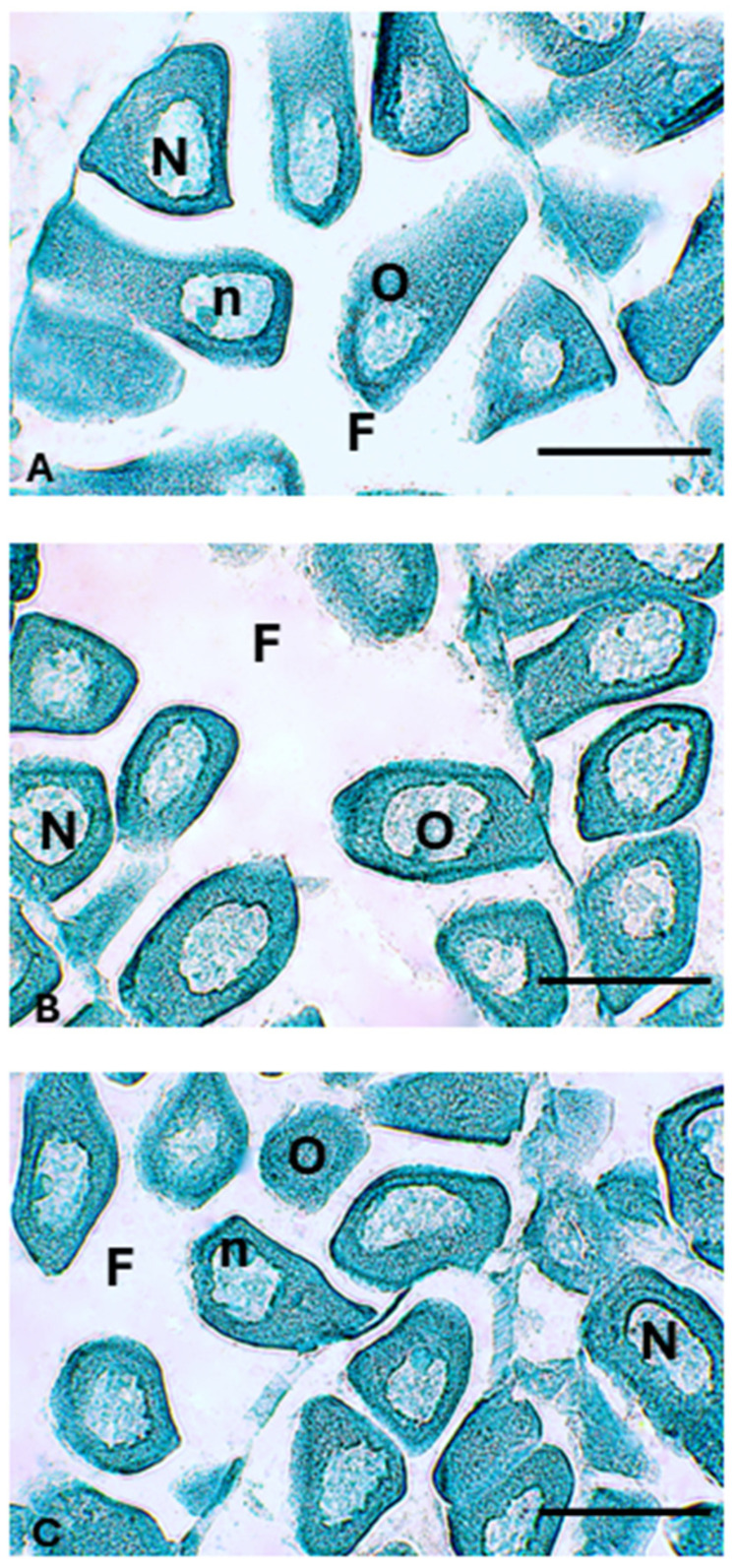
Representative histological sections of female gonads of *M. galloprovincialis* treated with Schmorl’s histochemical method showing no positivity in samples from (**A**) control, (**B**) exposed to low concentration of PS MPs (0.5 µg/mL), and (**C**) exposed to high concentration of PS MPs (1 µg/mL) group. Follicle (F), nucleus (N), nucleolus (n), oocyte (O). Scale bar: 20 μm.

Regarding the application of Schmorl’s method to the female gonads of mussels, no positive reaction was observed at any of the tested concentrations of PS MPs (0.5 µg/mL and 1 µg/mL), nor in the control group (Figure 4).

### 2.3. Western Blot for Proteins Involved in Cellular Stress

**Figure 5:**
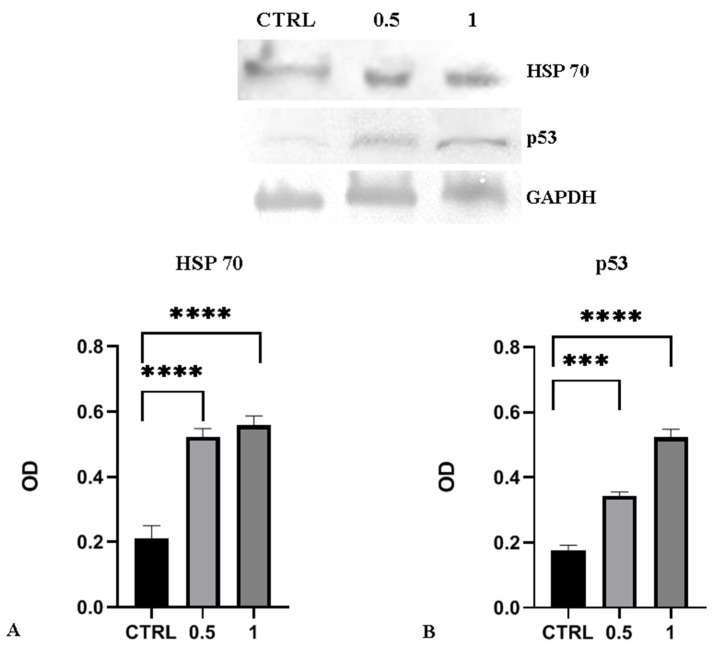
Expression of HSP70 and p53 proteins in ovarian tissues of *M. galloprovincialis*. Top panel: Representative western blot of protein extracts incubated with polyclonal anti-HSP70 and anti-p53. Bottom panel: Quantitative densitometric analysis of the protein bands, normalized in relation to GAPDH levels. Values are means ± SEM of three independent experiments. Statistical analysis was performed by ANOVA test with Bonferroni corrections. Asterisks indicate statistically significant differences compared to control cells: *** *p* < 0.001; **** *p* < 0.0001.

To further investigate the stress response triggered by the treatments, the expression levels of HSP70 and p53 were assessed in ovarian tissue from control and polystyrene MP-exposed animals using Western Blot analysis, followed by densitometric quantification. GAPDH served as the internal loading control for normalization. The analysis revealed a marked increase in both HSP70 and p53 protein levels in animals exposed to MPs. In detail, HSP70 increased significantly compared to the control for both MPs concentrations tested; the increase in p53 was dose-dependent (Figure 5).

### 2.4. Determination of Oxidative Stress

**Figure 6:**
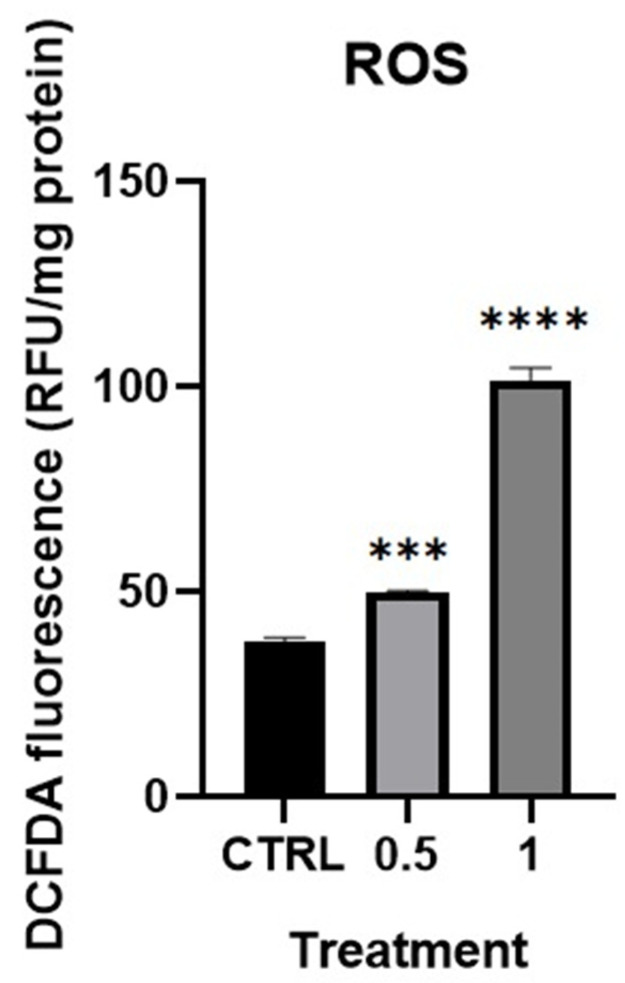
ROS levels in the female gonads of the mussel *M. galloprovincialis* after 48 h of exposure to low (0.5 μg/mL) and high (1 μg/mL) concentrations of PS MP, and in the control group (Ctrl). Statistical analysis was performed by ANOVA test with Bonferroni corrections. Asterisks indicate statistically significant differences compared to control cells: *** *p* < 0.001; **** *p* < 0.0001.

The analysis of the samples exposed to MPs revealed the increase in the ROS production with statistically significant differences compared to the control group (Figure 6). The levels of ROS increased in a concentration-dependent manner with increasing MPs concentrations, showing a distinct response among the experimental conditions. Such a trend was observed uniformly across the exposed groups, with the highest levels of ROS production being observed at the highest concentrations of exposure tested (Figure 6).

### 2.5. Determination of Glycosylated Proteins

**Figure 7:**
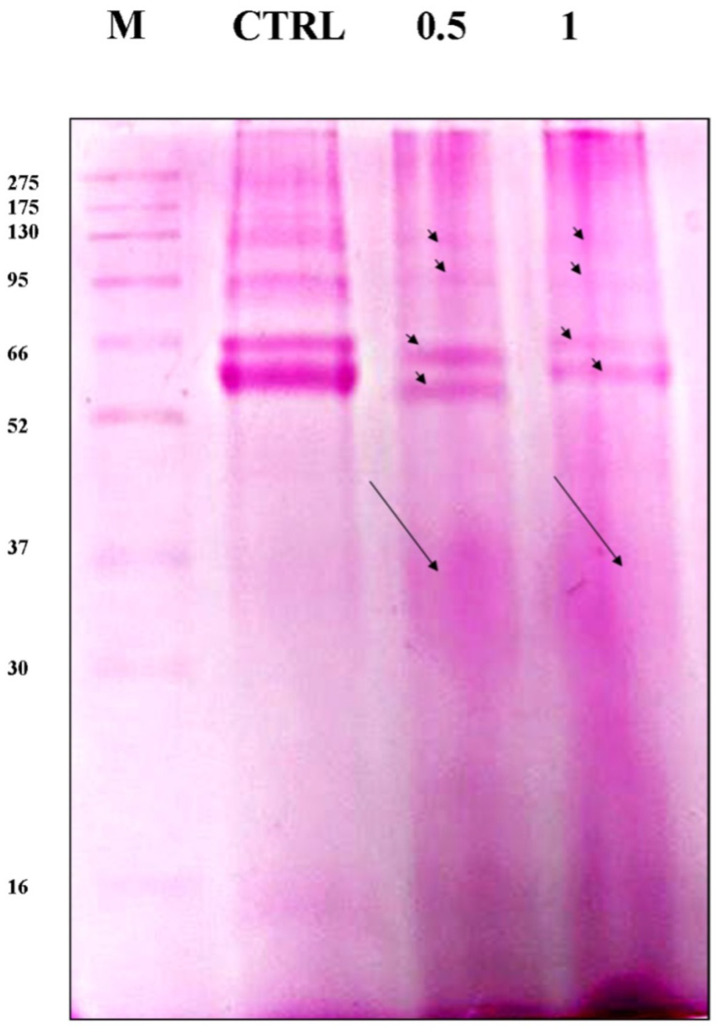
The effects of exposure to two concentrations of MPs (0.5 and 1 μg/mL) on the glycosylation of proteins extracted from *M. galloprovincialis* ovaries were examined using PAS. The arrowheads indicate bands with decreased intensity, and the arrow indicates the protein smear.

Exposure to MPs also induced time-dependent changes in protein glycosylation. In the control samples, the bands between 275 and 66 kDa were more intense, while in the samples exposed to MPs, a significant and dose-dependent reduction in their intensity was observed (Figure 7). Furthermore, in the lines corresponding to proteins extracted from the ovaries of animals exposed to MPs, the presence of protein smears was detected, indicating an alteration in the protein profile as well as glycosylation.

### 2.6. Metabolomic Data

**Figure 8:**
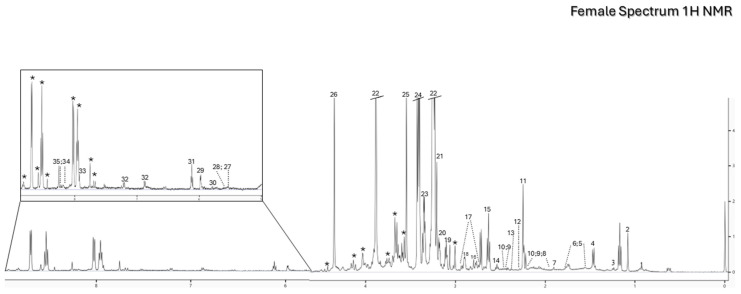
Representative ^1^H 1D 500 MHz NMR spectrum of the *M. galloprovincialis* female gonads from the control group showing the aliphatic region and, within the box, a vertical expansion of the aromatic region. Keys: 1-BCAAs (Leucine, Isoleucine, Valine), 2-Mytilitol, 3-Lactate, 4-Alanine, 5-Arginine, 6-Lysine, 7-Acetate, 8-Proline, 9-Glutamate, 10-Glutamine, 11-Acetoacetate, 12-Pyruvate, 13-Succinate, 14-βAlanine, 15-Methionine, 16-Sarcosine, 17-Aspartate, 18-N,N-Dimethylglycine, 19-Malonate, 20-Choline, 21-O-Phosphocholine, 22-Betaine, 23-Theophylline, 24-Taurine, 25-Glycine, 26-Homarine, 27-Glucose, 28-Glycogen, 29-UDP Glucose, 30-Uracil, 31-ATP/ADP, 32-Tyrosine, 33-Histidine, 34-IMP, 35-NADH/NADPH, *-Unknown.

The one-dimensional (1D) ^1^H NMR spectra obtained from metabolomic analysis of female gonads of *M. galloprovincialis* belonging to the control group showed the presence of the main polar metabolites (Figure 8). In particular, amino acids (branched-chain amino acids (BCAAs), arginine, etc.), osmolytes (betaine), neurotransmitters (choline), metabolites involved in energy metabolism (glucose) and tricarboxylic acid cycle intermediates (malonate) were identified within the metabolome of mussel female gonads. 

**Figure 9:**
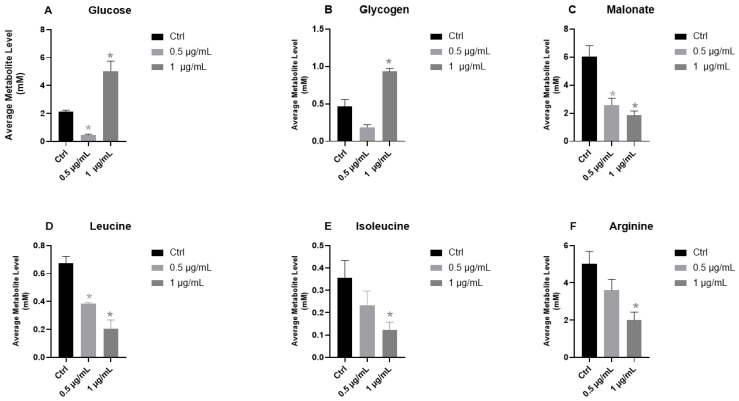
Graphs showing concentrations (mM, expressed as means) of (**A**) glucose, (**B**) glycogen, (**C**) malonate, (**D**) leucine, (**E**) isoleucine, and (**F**) arginine in the female gonads of the mussel *M. galloprovincialis* after 48 h of exposure to a low (0.5 μg/mL) and high (1 μg/mL) concentration of PS MPs and in the control group (Ctrl). Statistical analysis was performed by Welch’s one-way ANOVA, followed by the Games–Howell post hoc test for pairwise comparisons among experimental groups (0.5 µg/mL, 1 µg/mL) with respect to the control group (Ctrl), as indicated with asterisks (*), and with line-asterisks when compared to each other (0.5 µg/mL vs. 1 µg/mL). Statistical significance was set at q-value < 0.05.

By comparing the ^1^H NMR-1D spectra of gonadal samples treated with PS MPs with those obtained from mussels of the control group, metabolic differences were identified indicating that exposure to micro-polymers affected the metabolism of female mussels, particularly the energy pathways (glucose, glycogen, malonate) and protein turnover (BCAAs, arginine) (Figure 9). More specifically, in the female gonads of *M. galloprovincialis*, after 48 h of exposure to PS MPs, a marked decrease in glucose content was observed at the lowest concentration (0.5 μg/mL), while a significant increase was found at the highest concentration (1 μg/mL) (Figure 9A). Similarly, the relatively low glycogen energy reserves in the control group showed a trend like that of glucose (Figure 9B). Malonate showed a significant, dose-dependent downward trend (Figure 9C). Exposure to PS MPs also affected the amino acid metabolism, namely BCAAs and other free amino acids, such as arginine, that exhibited a dose-dependent reduction in their levels, with statistically significant differences (*p* < 0.05) especially in the group exposed to the highest concentration of PS MPs (Figure 9D).

**Figure 10:**
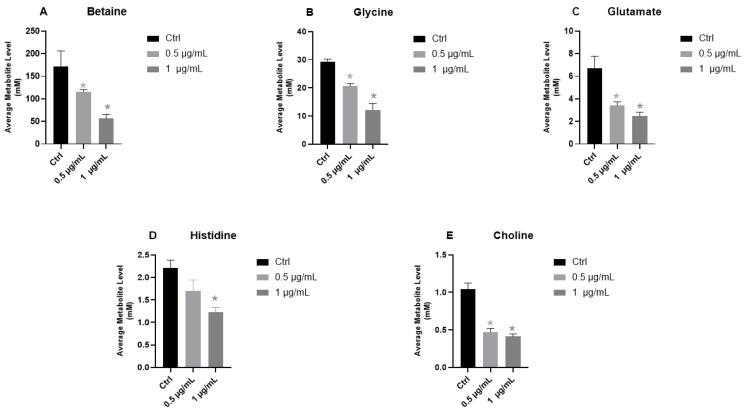
Graphs showing concentrations (mM, expressed as means) of (**A**) betaine, (**B**) glycine, (**C**) glutamate, (**D**) histidine, and (**E**) choline in the female gonads of the mussel *M. galloprovincialis* after 48 h of exposure to a low (0.5 μg/mL) and high (1 μg/mL) concentration of PS MPs and in the control group (Ctrl). Statistical analysis was performed by Welch’s one-way ANOVA, followed by the Games–Howell post hoc test for pairwise comparisons among experimental groups (0.5 µg/mL vs. 1 µg/mL) in respect to the control group (Ctrl), as indicated with asterisks (*), and with line-asterisks when compared to each other (0.5 µg/mL vs. 1 µg/mL). Statistical significance was set at q-value < 0.05.

Interestingly, betaine, which also serves as an osmolyte in mussel gonads, showed a monotonic and dose-dependent response, characterized by a significant decrease in its concentration (Figure 10A). Analysis of detected polar metabolites also revealed alterations in the levels of glycine and glutamate, two amino acids that are precursors of the tripeptide glutathione (GSH). Both showed a significant dose-dependent decrease in female gonads (Figure 10B,C). Histidine, a constituent of the detoxifying peptide ovothiol, indicated a trend towards depletion following exposure to PS MPs (Figure 10C), with an appreciable reduction already at the lowest concentration and a statistically significant depletion at the highest concentration of PS MPs. Finally, choline, an essential component of membrane phospholipids and a precursor of acetylcholine, showed a significant and dose-dependent reduction compared to the control group (Figure 10D).

Table 1 summarizes the changes measured in each metabolite mentioned, reporting the increase (↑) and decrease (↓) in its level as compared to the control group.

## 3. Discussion

MPs are now one of the main concerns in environmental research and marine ecosystem health. Their widespread and persistent presence in surface waters and coastal sediments has led the scientific community to question more urgently the effects that these emerging contaminants may have on marine fauna and, more generally, on the balance and sustainability of aquatic ecosystems [21,22]. These particles are not only a potential vector for toxic chemicals and pathogenic microorganisms [23] but can also directly interfere with the physiological and cellular processes of aquatic organisms, compromising vital functions such as reproduction [19].

In light of these considerations, assessing the impact of MPs on marine fauna has become a fundamental priority in order to fully understand the ecotoxicological consequences of this form of pollution. Studies of the reproductive systems of bivalves are generally underrepresented due to the complex biological challenges involved, such as the wide variety of reproductive strategies employed, including sequential or simultaneous hermaphroditism. Furthermore, ecotoxicological studies on edible mollusks tend to focus on verifying the accumulation of contaminants rather than on the effects on reproductive health of organisms exposed to contaminants. In this perspective, the present study investigated the effects induced by exposure to polystyrene MPs in female specimens of *Mytilus galloprovincialis*, a bivalve mollusk widely distributed in the Mediterranean Sea and recognized as an ideal bioindicator for ecotoxicological studies. The analysis was conducted at both histological and metabolic levels to obtain an integrated view of the structural and functional alterations related to exposure.

Based on histological observations, a significant increase in morphological and structural damage to the ovaries of mussel females exposed to MPs was detected. The alterations by Hematoxylin–Eosin staining evidenced a marked reduction in the number of oocytes and changes in both oocyte and follicle structure. In treated samples, there was a greater presence of interfollicular empty spaces and vitellogenic oocytes in degeneration, with increasing intensity in the groups exposed to concentrations of 1 μg/mL of PS MPs. In a recent study conducted on males, it has been demonstrated that treatment with MPs at the same concentrations induced an increase in oxidative stress [19]. Exposure to environmental contaminants induces an increase in the production of reactive oxygen species (ROS), which can exceed the efficiency of cellular antioxidant systems, leading to a redox imbalance [24]. Oocytes, due to their high lipid content and intense metabolic activity associated with gamete maturation, are particularly vulnerable to oxidative damage, which manifests as lipid peroxidation, mitochondrial alterations, and DNA damage. These processes have been associated with cytoplasmic disorganization, oocyte atresia and reduced gamete quality [24,25,26]. The dose-dependent stress condition induced by MPs was confirmed by the presence in all treated animals of PAS-positive hypertrophic cup-shaped cells located in the peripheral part of the mantle. According to the literature, the appearance of these cells in mussel tissues can be interpreted as an indicator of increased production of glycoproteins and polysaccharides, molecules known for their protective and structural role [27]. This response can be considered a compensatory mechanism aimed at safeguarding cellular homeostasis and reducing tissue damage induced by MPs. The state of cellular stress was further confirmed by biochemical analysis of protein gels, performed using PAS. This analysis showed a marked reduction in band intensity in the treated samples compared to the controls, indicating an overall decrease in glycoprotein content. This data suggests an alteration in the processes of glycosylation and/or synthesis of proteins destined for the secretory pathway. In accordance with what has been widely reported in the literature, MPs are capable of inducing stress in the endoplasmic reticulum, a key cellular compartment for the synthesis, folding and maturation of glycoproteins [28,29]. Impaired endoplasmic reticulum function may therefore be reflected in reduced secretory pathway efficiency, contributing to the biochemical alterations observed in the treated samples. 

The structural alterations found in treated animals were herein supported by another biochemical investigation. Specifically, cellular exposure to MPs at concentrations of 0.5 and 1 µg/mL resulted in increased expression levels of HSP70 and P53 proteins, showing a dose-dependent trend. The increase in HSP70 is consistent with the activation of the proteotoxic stress response, typical of conditions in which the accumulation of misfolded proteins or the increase in ROS induces the expression of heat shock proteins [30]. At the same time, the upregulation of p53 suggests the activation of cellular damage response pathways, a phenomenon frequently observed when pollutants generate oxidative stress or genomic instability [31].

The dose-dependent trend observed is in line with what has been reported in the literature: increasing exposure to MPs or NPs can amplify oxidative stress and proportionally modulate the activation of genes associated with proteostasis and cell survival [32,33,34,35]. Oxidative stress was confirmed by direct ROS measurement, where we observed an increase in reactive oxygen species in the exposed samples compared with the controls, with a dose-dependent pattern. These results therefore confirm that even short-term exposure to MPs is capable of triggering sensitive and measurable responses, with potential biological implications, especially in tissues characterized by high susceptibility to environmental stress.

Several studies confirm that MPs and NPs can influence the cellular and humoral immune response and, consequently, alter defenses against other environmental stressors, threatening the health of marine invertebrates such as *M. galloprovincialis* on multiple fronts [35,36,37]. Invertebrate hemocytes are classified into various classes depending on their morphology and presence of granules within them. Indeed, they are distinguished by their size and presence/absence of cytoplasmic granules. In this regard, cells can be defined as granulocytes, with a low nucleus/cytoplasm (N/C) ratio and granules containing hydrolytic and/or oxidative enzymes, and as agranulocytes/hyalinocytes, with a high N/C ratio but without the presence of granules. These are also added to subclasses based on their responsiveness to dyes, and therefore identified as acidophilic, basophilic, and mixed [35,38]. The great variety reflects the various functions they perform, such as the immune response against pathogens, performing phagocytic activity, encapsulation with melanin deposits through the cascade of biochemical reactions of the phenoloxidase system, production of ROS and signaling molecules such as cytokines, tissue and shell repair, and reproduction [39,40,41,42]. It has been demonstrated that PS MPs can compromise chemotactic activity in the red clam *Tegillarca granosa*, determining a 76.28% decrease in the hemocytes migration rate [43]. This finding is consistent with the decrease in cell counts of all three classes observed in female gonads exposed to treatment with 0.5 µg/mL and 1 µg/mL of PS MPs, an effect possibly promoted by a decrease in the migration capacity of hemocytes within the gonadal tissue. This condition could pose a threat to the responsiveness to other external xenobiotics for benthic, sessile, and filter-feeding organisms such as bivalves. Furthermore, hemocytes can produce a high amount of ROS through the activity of dual oxidase-2 (Duox-2) (mainly hydrogen peroxide), which plays a fundamental role in the antibacterial response [44]. However, if not quickly eliminated, the ROS can damage the redox balance. In this context, it does not surprise us to have found a dose-dependent decrease in the amino acid histidine, precursor of ovothiol, scavenger of hydrogen peroxide [45], and glycine and glutathione, constituents of the tripeptide glutathione (GSH) involved in detoxification processes, following exposure to MPs. These data are in line with what occurred in the gills of *M. galloprovincialis* after a 72 h exposure to 3 µm PS MPs [46]. The lack of melanin deposits suggests that the short exposure time was not sufficient to activate the opsonizing capacities that would lead to melanin production by the phenoloxidase system [47], as well as the altered presence of hemocytes compared to the control group could be evidence of the decline of downstream pathways, related to the immune system and compromised by the exposure to MPs [43].

Capolupo et al. [13] reported that in filter-feeding species, such as *M. edulis*, the ingestion of microparticles can limit filtration dynamics. This, as observed in other species exposed to MPs [48], results in the reduction of key metabolites of the energy pathway, such as glucose and glycogen, which are fundamental to basal metabolism and of great importance within the reproductive system, being important to the spawning event and correct gametogenesis, mainly in the female gonad, since oocytes are responsible for conserving reserves for the growth of the future embryos [49,50]. The finding of a biphasic response for glucose and glycogen raised interesting questions. Comparing the two treatment groups (0.5 µg/mL vs. 1 µg/mL), relevant significance emerged for both glucose and glycogen. This reinforced the idea that the energy pathway supported by these two metabolites and involving the glycosidic and glycogenic pathways is influenced in a dose-dependent manner by PS MPs. The biphasic glucose/glycogen response would suggest a hormetic event. However, this remains a hypothesis as only two doses of microparticles were tested (0.5 µg/mL and 1 µg/mL), and therefore, it is difficult to validate the data from this perspective, which requires further investigation. What can be confirmed is that, depending on the treatment dose, female gonads respond differently to the same stressor, exhausting energy resources at low concentrations (0.5 µg/mL) and implementing compensatory mechanisms in response to high concentrations (1 µg/mL). This is in line with what has been documented in similar species such as *M. coruscus* exposed to PS MPs [51]. Also, Capolupo et al. [13] suggested that the different data recorded following two different treatments may depend on a hormetic response to the diverse doses used, highlighting a different modulation of adaptive capacities in presence of increasing stress factors. However, the dose-dependent decrease in malonate, a competitive inhibitor of the enzyme succinate dehydrogenase, crucial in the energy pathway linked to the tricarboxylic acid cycle, seems to indicate a shift towards fermentative mechanisms to compensate for a greater energy demand in response to other stress factors [17,52]. This latter finding could be justified by a compensatory mechanism represented by a greater protein turnover, as evidenced by a dose-dependent decrease in leucine and isoleucine, as well as arginine. Alterations in the metabolism of BCAAs, histidine and the biosynthesis of osmolytes such as taurine were also highlighted in other bivalves such as *Crassostrea gigas* exposed to higher MPs concentrations (in the order of mg/L), underlining that such upheavals may lead to a greater susceptibility to dysfunctions and oxidative stress [53].

Considering what was observed for the male counterpart, on which a previous study was conducted under the same experimental conditions [19], it is possible to state that the adaptive capacities to an insult from PS MPs are evidently influenced by sexual character. However, some findings are mutually consistent, perhaps due to the unique role played by basal and structural mechanisms of the reproductive system. One example is represented by choline, which undergoes a significant dose-dependent decrease in both male and female gonads. This is also confirmed by the finding of a similar response in a different organ such as the gills, where neurotransmission mechanisms are fundamental for cilia motility [46]. Therefore, the adaptation could be directly linked to the structure and functioning of the gonad, since cholinergic and monoaminergic regulatory systems are present as found in the gonads of *Mytilus unguiculatus* [54,55]. It is also possible that compensatory mechanisms are implemented in the female gonad that modulate the concentration of the two metabolites betaine and choline. In fact, it has been suggested that in the female gonads of *M. galloprovincialis,* choline and betaine are strictly interdependent [56]. The synthesis of betaine is dependent on the oxidation of choline, and a decrease in one could affect the biosynthesis of the other, and this would compromise the protective action of betaine against catalase enzymes, increasing the risk of oxidative damage to the plasma membrane [57].

The data prove that exposure to PS MPs can alter the normal functioning of the reproductive system, causing repercussions on the health of organisms such as edible invertebrates [58].

## 4. Materials and Methods

### 4.1. Experimental Design

In December 2022, during the breeding season, sexually mature specimens of *M. galloprovincialis* were collected in Bacoli (Naples, Italy) and transferred to the Stazione Zoologica Anton Dohrn in Naples, where they were stabled and acclimatized for 15 days at a temperature of approximately 18 °C with filtered seawater recirculation. The valves were approximately 7 cm long and randomly assigned to 30-litre tanks (n = 30/tank) with closed water recirculation for subsequent MPs treatments. The experimental design involved exposing the mussels for 48 h to two concentrations of polystyrene (PS) MPs (0.5 μg/mL and 1 μg/mL), using PS-R-5.0 (10% *w*/*v*, referred to as MP-pristine) with a diameter of 5 μm (MicroParticles GmbH, Berlin, Germany). The control group was kept in the same conditions but without the addition of the MPS. Three tanks, representing independent replicates, have been set up for each experimental set; 6 females were taken from each tank, making a total of 18 animals per experimental point. Following treatment, the female gonads of each animal were collected; part of the tissue was used for histological and histochemical investigations and thus fixed in Bouin’s solution, while the remaining part of the gonad was stored at −80 °C for biochemical and metabolomic investigations. Sex was preliminarily determined by preparing a fresh tissue smear on a glass slide and performing a rapid microscopic examination; definitive confirmation was subsequently obtained through histological analysis of all collected samples. Statistical analyses were carried out on the animals from the three distinct tanks for each experimental point, treating each animal as an independent replicate regardless of the tank from which it originated.

### 4.2. Histological and Histochemical Analyses

Ovarian samples were taken from 10 mussels in each experimental group and fixed in Bouin’s solution for 24 h, then dehydrated in a series of increasing concentrations of alcohol and embedded in paraffin for histological analysis. Sections of 5 µm were stained with Hematoxylin–Eosin to evaluate the general morphology of the gonads [59]. PAS reaction was performed on the same tissues to highlight mucin, glycogen and glycoproteins. Other sections were treated with the May–Grünwald Giemsa reaction (Bio-Optica Kit, 04-081802) to highlight the presence of different hemocyte classes within the gonads, and with the Schmorl’s method, applied according to the revised protocol of Shataer et al. [60] by employing ferric chloride 3% and potassium ferrocyanide 1% in ratio 1:1, to verify any melanin deposits, as the final product of the phenoloxidase enzyme. Morphological observations were made using a Zeiss Axioskop microscope equipped with Axiocam version 305 and Zen version3.8 software [61], available at the Advanced Microscopy Core of the Department of Biology, University of Naples Federico II.

### 4.3. Western Blot Analysis

For Western Blots analysis of proteins involved in cellular stress, small pieces of ovaries (n = 6) were manually homogenized in a Potter mortar at 4 °C in 5 mL of RIPA lysis buffer (50 mM Tris, 150 mM NaCl, 0.1% SDS, 0.5% sodium deoxycholate, 5 mM NaF, 1% NP-40, 10 mM EDTA) containing a protease inhibitor cocktail (Sigma-Aldrich, St. Louis, MO, USA) previously [62]. The homogenate was centrifuged at 7000× *g* for 10 min at 4 °C and the proteins were quantified using the PIERCE method. Fifty μg of protein was loaded onto a 13% SDS-polyacrylamide gel at 60 mA, and then stained with Coomassie Blue R-250. The proteins were then transferred to a nitrocellulose membrane at 350 mA for 1 h. The membrane was blocked at room temperature first with 7% milk powder in 1× TBS-Tween for 45 min, then with 7% BSA in 1× TBS-Tween for another 45 min. Subsequently, it was incubated overnight at 4 °C with rabbit anti-HSP70 primary antibody (Elabsciences, E-AB-92322, 1:1000) in TBS-Tween + 2% milk and rabbit anti-p53 primary antibody (Elabsciences, E-AB-32469, 1:300) in TBS-Tween + 2% milk. The following day, the membrane was washed in TBS-Tween and incubated for 1 h with peroxidase-conjugated anti-rabbit IgG secondary antibody (Santa Cruz, Sc-2005, 1:2000) in TBS-T + 2.5% milk. Rabbit anti-GAPDH antibody (Elabscience, E-AB-40516, 1:2000) was used to normalize the protein transfer. For quantitative detection, protein bands were detected with chemiluminescent HRP substrate (Amersham, Thermo Fisher Scientific, Milan, Italy) and visualized using a ChemiDoc Image System scanner (Bio-Rad Laboratories, Hercules, CA, USA). The optical density (OD) of the bands was analyzed with ImageJ 1.54F, and the results were graphically represented with GraphPad 5.0 (San Diego, CA, USA). To detect glycosylated proteins, unstained gels were incubated for 15 min in a 0.5% periodic acid solution, washed in distilled water, and stained with Schiff’s reagent for 5 min in the dark. The gels were decolored in 0.5% sodium metabisulphite until the bands appeared clearly.

### 4.4. 2′,7′-Dichlorofluorescein Diacetate (DCFDA) Fluorescent ROS Detector Probe 

ROS levels were evaluated using a fluorimetric assay with the fluorescent probe 2’,7’-dichlorofluorescein diacetate (DCFDA). Approximately 1 g of fresh tissue from the control and treatment samples (n = 6) was collected and ground into a fine powder in liquid nitrogen. Each tissue powder sample was weighed and transferred to 2 mL tubes containing 1.5 mL of lysis buffer. The suspension was then homogenized using a TissueLyser with tungsten beads for 10 min at 40 Hz. The obtained extracts were then centrifuged at 6500 rpm for 30 min at 4 °C, after which the supernatant was subjected to an additional centrifugation step at 12,000× *g* for 10 min at 4 °C to remove any possible cell debris. ROS production was measured by incubating 10 µL of the supernatant with 90 µL of a buffer containing 1 mM DCFDA (final DCFDA concentration 10 µM). The mixture was thoroughly mixed and incubated in the dark for 10 min. The values obtained for each sample were corrected for background fluorescence by measuring a blank sample consisting of 10 µL of supernatant and 90 µL of buffer without DCFDA. Fluorescence was measured using excitation and emission wavelengths of 485 and 528 nm, respectively. The values obtained were expressed as relative fluorescence units (RFU). Total proteins in the extracts were quantified using the PIERCE method. ROS levels were expressed as ROS = RFU/mg protein, where RFU is the fluorescent signal corrected for the blank and mg of protein is the protein concentration determined by the PIERCE method. 

### 4.5. Metabolomic Analysis 

#### 4.5.1. Extraction of Metabolites from the Ovary of *M. galloprovincialis*

To obtain a complete picture of the reproductive health of female mussels, a metabolomic analysis was conducted according to the protocol described previously [63]. To this end, sub-samples of approximately 100 mg of gonadal tissue were taken and subsequently subjected to metabolite extraction using a two-step protocol (methanol/chloroform/water). After homogenizing the samples (n = 5) with stainless steel beads (diameter 3.2 mm) in the presence of a mixture of cold methanol and distilled water (4 mL/g: 0.85 mL/g) using a TissueLyser LT (Qiagen, Hilden, Germany) for 10 min, chloroform (4 mL/g) and distilled water (2 mL/g) were added. The samples were then shaken, kept briefly on ice and centrifuged at 2000 *g* for 10 min at 4 °C, achieving separation between the polar and non-polar phases. From the polar phase, containing the hydrophilic metabolites, 600 μL of supernatant was collected, transferred to clean tubes, and subsequently dried in a vacuum centrifugal concentrator (Eppendorf 5301, Milan, Italy) until a completely dry pellet was obtained, which was stored at −20 °C until NMR analysis. Prior to spectral acquisition, each sample was resuspended in 0.1 M sodium phosphate buffer (600 μL; pH 7.0; containing 10% D_2_O; Armar AG, Döttingen, Switzerland) and 1 mM 2,2-dimethyl-2-silapentano-5-sulfonate (DSS) (Sigma-Aldrich), used as an internal standard for chemical calibration. The suspension of polar metabolites was then carefully transferred to glass capillaries for NMR, avoiding the formation of air bubbles to ensure sample homogeneity and the accuracy of the subsequent spectroscopic analysis.

#### 4.5.2. Metabolomics Based on ^1^H NMR and Spectral Pre-Processing 

One-dimensional (1D) ^1^H NMR spectra were acquired using a Varian-500 NMR spectrometer equipped with RF channels and waveform generators, operating at a frequency of 499.74 MHz and a temperature of 298 K, by applying the 1D Nuclear Overhauser Effect SpectroscopY (NOESY) pulse sequence (noesypr1d) with pre-saturation during the 120 ms mixing time and 2 s relaxation delay for water suppression. For each mussel gonad spectrum, a total of 32 transients were collected in 32 k data points over a spectral width of 6009 Hz using a recycle time of 3.5 s and a mixing time of 1 ms. After Fourier transformation with 0.5 Hz line broadening, manual phasing of each spectrum was performed using Chenomx Processor software (Chenomx NMR Suite v11.0; Chenomx Inc., Edmonton, AB, Canada), applying baseline correction and setting the DSS signal as a chemical reference at 0.00 ppm. Metabolite identification and quantification were performed using the Chenomx 500 MHz database and other public computer libraries. Subsequently, using the Chenomx Profiler module (Chenomx NMR Suite), the spectra were segmented into chemical shift intervals of 0.005 ppm (ranging from 0.8 to 8.8 ppm). Finally, the data were normalized as previously reported [21].

### 4.6. Statistical Analysis

Histochemical data were manually quantified and subsequently processed in Microsoft Excel and GraphPad Prism (version 8.0; San Diego, CA, USA). Differences between groups were analyzed using two-way ANOVA, considering treatment (Ctrl, 0.5 µg/mL, 1 µg/mL) and hemocyte class (acidophils, basophils, and mix) as factors, followed by the Holm–Šídák post hoc test for multiple comparisons between treatments and the control group within each cell class. Statistical significance was set at *p* < 0.05. For metabolomic data obtained from qualitative and quantitative analysis performed with Chenomx software (Chenomx NMR Suite version 12.0 professional; Chenomx Inc., Canada), data were exported to Microsoft Excel for calculation of mean values and standard deviation (± SD), and subsequently analyzed with GraphPad Prism (version 8.0; San Diego, CA, USA). Homogeneity of variance was assessed using the Brown–Forsythe test, and in the presence of heteroskedasticity, differences between groups were analyzed using Welch’s one-way ANOVA, followed by the Games–Howell post hoc test for pairwise comparisons among all experimental groups (Ctrl, 0.5 µg/mL, and 1 µg/mL). To account for multiple testing of metabolites, *p*-values were corrected using the Benjamini–Hochberg procedure to control for the false discovery rate (FDR). Statistical significance was set at q-value < 0.05. Finally, Western blot and DCFDA data were analyzed using an ANOVA test with Bonferroni correction and *p*-value < 0.05 was considered significant. 

## 5. Conclusions

In conclusion, the results obtained in this study show that, in *M. galloprovincialis* females, a short-term (48 h) exposure to polystyrene MPs causes significant effects on gonadal tissues, both at histological, biochemical, and metabolic levels. Histological differences observed include degenerating oocytes, hypertrophy of mucus-producing cells, and changes in hemocyte types. At the biochemical level, changes in protein glycosylation and increased expression of stress proteins, such as HSP70 and p53, are other unequivocal changes observed following exposure to MPs. Finally, the observation of a compromised metabolic profile suggests the activation of immediate defense mechanisms against oxidative stress induced by MPs. Indeed, similar to what has been observed in males [19], polystyrene MPs had a marked effect on the energy and amino acid metabolism of females, albeit with a different pattern. In particular, the alterations found in glucose and glycogen levels, associated with changes in the metabolism of BCAAs and arginine, indicate a disruption of the main bioenergetic pathways. However, compared to males, in whom a compensatory response was observed, exposure in females led to a progressive consumption of energy reserves, likely linked to the high metabolic expenditure required during gamete maturation and deposition.

This condition suggests that, in females, the interference of MPs with normal metabolic processes not only compromises the efficiency of the bioenergetic cycle but may also affect the vitality and quality of germ cells, hindering the successful outcome of external fertilization. These results, read in parallel with those already reported for males [19], confirm the presence of a sexually dimorphic response to the effects of polystyrene MPs, highlighting the great vulnerability of both *M. galloprovincialis* males and females to metabolic stress induced by these emerging contaminants.

## Figures and Tables

**Table 1:** Metabolites detected by ^1^H NMR-based metabolomics within the female gonads of mussel *Mytilus galloprovincialis*, alongside their relative chemical shift and peak shape, and their percentage variation in response to 0.5 µg/mL and 1 µg/mL of PS MPs in respect to the control group (↑: increase; ↓: decrease).

Metabolites Involved	Chemical Shift and Peak Shape (ppm)	CHANGE IN%	
		0.5 µg/mL	1 µg/mL
*Amino Acid Metabolism*		48 h	48 h
Arginine Glycine Glutamate Histidine Isoleucine Leucine Choline	3.8 (t), 3.2 (t), 1.9 (m), 1.7 (m) 3.6 (s) 3.8 (q), 2.4 (m), 2.1 (m) 4.0 (m), 3.2 (q), 3.1 (m) 3.7 (d), 1.0 (d), 0.9 (t) 3.7 (m), 1.7 (m), 1.0 (t) 4.1 (m), 3.2 (s)	↓ 17 ↓ 27 ↓ 49 ↓ 19 ↓ 18 ↓ 15 ↓ 55	↓ 46 ↓ 57 ↓ 71 ↓ 27 ↓ 43 ↓ 52 ↓ 61
*Energy Metabolism*			
Glycogen Glucose Malonate	5.4 (s), 3.8 (m), 3.6 (m), 3.4 (m) 5.2 (d), 3.8 (m), 3.7 (m), 3.5 (m) 3.1 (s)	↓ 49 ↓ 60 ↓ 44	↑ 150 ↑ 168 ↓ 62
*Osmoregulation*			
Betaine	3.9 (s), 3.3 (s)	↓ 11	↓ 57

## Data Availability

The original contributions presented in this study are included in the article. Further inquiries can be directed to the corresponding authors.
